# Heterogeneity of treatment effect of vilobelimab in COVID-19: a secondary analysis of a randomised controlled trial

**DOI:** 10.1186/s13054-024-05004-z

**Published:** 2024-06-28

**Authors:** Rombout B. E. van Amstel, Marleen A. Slim, Endry H. T. Lim, Simon Rückinger, Christopher W. Seymour, Bruce P. Burnett, Lieuwe D. J. Bos, Lonneke A. van Vught, Niels C. Riedemann, Diederik van de Beek, Alexander P. J. Vlaar, the PANAMO Study Group, Martin Witzenrath, Pieter van Paassen, Leo M. A. Heunks, Bruno Mourvillier, Sanne de Bruin, Matthijs C. Brouwer, Pieter R. Tuinman, José Francisco K. Saraiva, Gernot Marx, Suzana M. Lobo, Rodrigo Boldo, Jesus A. Simon-Campos, Alexander D. Cornet, Anastasia Grebenyuk, Johannes M. Engelbrecht, Murimisi Mukansi, Philippe G. Jorens, Robert Zerbib, Korinna Pilz, Renfeng Guo, Pierre Bulpa, Fabio S. Taccone, Greet Hermans, Marc Diltoer, Michael Piagnerelli, Nikolaas De Neve, Antonio T. Freire, Felipe D. Pizzol, Anna Karolina Marinho, Victor H. Sato, Clovis Arns da Cunha, Mathilde Neuville, Jean Dellamonica, Djillali Annane, Antoine Roquilly, Jean Luc Diehl, Francis Schneider, Jean Paul Mira, Jean Baptiste Lascarrou, Luc Desmedt, Claire Dupuis, Carole Schwebel, Guillaume Thiéry, Matthias Gründling, Marc Berger, Tobias Welte, Michael Bauer, Ulrich Jaschinski, Klaus Matschke, Roberto Mercado-Longoria, Belinda Gomez Quintana, Jorge Alberto Zamudio-Lerma, Juan Moreno Hoyos Abril, Angel Aleman Marquez, Peter Pickkers, Luuk Otterspoor, Luis Hercilla Vásquez, Carlos Rafael Seas Ramos, Alejandro Peña Villalobos, Gonzalo Gianella Malca, Victoria Chávez, Victor Filimonov, Vladimir Kulabukhov, Pinak Acharya, Sjoerd A. M. E. G. Timmermans, Matthias H. Busch, Floor L. F. van Baarle, Rutger Koning, Liora ter Horst, Nora Chekrouni, Thijs M. van Soest, Rombout B. E. van Amstel, Sabine E. Olie, Ingeborg E. van Zeggeren, Marcel C. G. van de Poll, Claus Thielert, Dorothee Neukirchen

**Affiliations:** 1 Department of Intensive Care Medicine, Amsterdam University Medical Centers, University of Amsterdam, Meibergdreef 9, 1105 AZ Amsterdam, The Netherlands; 2 Center for Experimental and Molecular Medicine, Amsterdam University Medical Centers, University of Amsterdam, Amsterdam, The Netherlands; 3 Department of Neurology, Amsterdam University Medical Centers, University of Amsterdam, Amsterdam, The Netherlands; 4 Metronomia Clinical Research GmbH, Munich, Germany; 5 Department of Critical Care Medicine, School of Medicine, University of Pittsburgh, Pittsburgh, PA USA; 6 Department of Emergency Medicine, School of Medicine, University of Pittsburgh, Pittsburgh, PA USA; 7 Clinical Research, Investigation, and Systems Modeling of Acute Illness Center, School of Medicine, University of Pittsburgh, Pittsburgh, PA USA; 8 InflaRx Pharmaceuticals Inc, Ann Arbor, MI USA; 9 InflaRx, Jena, Germany

**Keywords:** COVID-19, Immunomodulation, Complement, Vilobelimab, Phenotype, Cluster analysis, Subtypes

## Abstract

In a phase 3 trial (PANAMO, NCT04333420), vilobelimab, a complement 5a (C5a) inhibitor, reduced 28-day mortality in mechanically ventilated COVID-19 patients. This post hoc analysis of 368 patients aimed to explore treatment heterogeneity through unsupervised learning. All available clinical variables at baseline were used as input. Treatment heterogeneity was assessed using latent class analysis (LCA), Ward’s hierarchical clustering (HC) and the adjudication to previously described clinical sepsis phenotypes. The primary outcome was 28-day mortality. For LCA, a 2-class latent model was deemed most suitable. In the LCA model, 82 (22%) patients were assigned to class 1 and 286 (78%) to class 2. Class 1 was defined by more severely ill patients with significantly higher mortality. In an adjusted logistic regression, no heterogeneity of treatment effect (HTE) between classes was observed (*p* = 0.998). For HC, no significant classes were found (*p* = 0.669). Using the previously described clinical sepsis subtypes, 41 patients (11%) were adjudicated subtype alpha (α), 17 (5%) beta (β), 112 (30%) delta (δ) and 198 (54%) gamma (γ). HTE was observed between clinical subtypes (*p* = 0.001) with improved 28-day mortality after treatment with vilobelimab for the δ subtype (OR = 0.17, 95% CI 0.07–0.40, *p* < 0.001). No signal for harm of treatment with vilobelimab was observed in any class or clinical subtype. Overall, treatment effect with vilobelimab was consistent across different classes and subtypes, except for the δ subtype, suggesting potential additional benefit for the most severely ill patients.

**Supplementary Information:**

The online version contains supplementary material available at 10.1186/s13054-024-05004-z.

## Introduction

A randomised, double-blind, placebo-controlled, multicentre phase 3 trial in mechanically ventilated patients with coronavirus disease 2019 (COVID-19) (PANAMO trial, NCT04333420) showed that vilobelimab, a monoclonal antibody which specifically binds complement 5a (C5a), reduced all-cause mortality at day 28 from 40 to 31% [1]. The pathophysiology of COVID-19 is an interaction between immune disturbances, endothelial dysfunction, and thromboembolic complications. This complex interaction varies between patients [2], and several clinicians and researchers advocate for a more personalized approach in the treatment of COVID-19 patients [3]. The large differences in host response are reflected through heterogeneity of treatment effect (HTE) of immunomodulatory agents [2].

Using four clinical subtypes previously described in patients presented at the emergency department (ED) with sepsis [4], survival benefit of the use of dexamethasone in COVID-19 patients was only seen in the subtype with the highest inflammation, also called the δ subtype [5]. A similar mortality benefit was observed in critically ill COVID-19 patients treated with corticosteroids and belonging to a different hyperinflammatory subtype [6]. Other immunomodulatory agents, such as imatinib, tocilizumab and anakinra, also showed HTE in COVID-19 [7–9]. Nowadays, however, subphenotypes have limited use in clinical practice, partly caused by the complexity in assigning subphenotypes. Using routinely available clinical variables limits this problem. Future studies investigating immunomodulation treatment in COVID-19 might benefit from using patient enrichment to identify those with better treatment response. Moreover, patient enrichment can identify subgroups that may experience adverse effects and can reduce costs by avoiding prescription in patients not likely to benefit from treatment.

In this post hoc analysis of a randomised controlled trial, the aim is to investigate the heterogeneity in vilobelimab’s treatment effect and adverse events in critically ill COVID-19 patients. Routinely measured clinical data was used to identify classes and to assign to known subtypes. We postulate that clusters and subtypes will exhibit differential treatment effect based on differences in inflammation between the clusters.

## Methods

### Study design and patient selection

This study was conducted as a secondary analysis of the PANAMO trial (NCT04333420) [1]. From October 1, 2020 to October 4, 2021, 369 critically ill COVID-19 patients were included from 46 hospitals in Europe, Africa and North- and South-America. Inclusion criteria were an age of 18 years or older, invasive mechanical ventilation within 48 h before the first infusion of study medication, a PaO_2_/FiO_2_ (PF-ratio) of 60–200 mmHg and a confirmed severe acute respiratory syndrome coronavirus 2 (SARS-CoV-2) infection in the past 14 days. The complete exclusion criteria can be found in the original report [1]. For the current analysis, 368 patients were included due to random assignment in error in one patient. Treatment emergent adverse events (TEAE) were defined as any event that occurred or worsened at or after the first infusion, with AE defined as any untoward medical occurrence in a patient or clinical study patient, temporally associated with the use of Investigational medicinal product (IMP), whether or not considered related to the IMP [1].

### Clustering techniques

All available clinical variables, such as vital signs and hematology, coagulation and chemistry laboratory measurements, were collected at baseline and used as input for unsupervised learning (Supplementary Table 1). The three techniques used were: (1) latent class analysis (LCA), (2) Ward’s hierarchical clustering (HC) and (3) the adjudication to clinical subtypes previously described in patients presented at the ED for sepsis (SENECA subtypes, Supplementary Table 6) [4]. LCA was conducted using the R package ‘Flexmix’. The process of model design and LCA followed the steps and considerations outlined by Sinha et al. [10], see the supplementary methods for more details. Next, Ward’s HC using Monte Carlo reference-based consensus clustering (M3C) was done [11]. M3C constructs a Monte Carlo *p*-value and Beta distribution *p*-value to test against the null distribution, which is the existence of a homogeneous cohort and thus no (statistical) clusters exist. The Relative Cluster Stability Index is an additional criterion. If significant classes were found, HTE was analyzed. Lastly, clinical variables were used to identify the subtypes alpha (α), beta (β), gamma (γ), and delta (δ) using the SENECA approach [4] as previously described [12]. In short, all the available variables (16 of the original 29 variables, Supplementary Table 6) were log-transformed (if needed), scaled, centered and used to assign the subtype by Euclidean distance. If variables were not completely missing, multivariate imputation by chained equations (MICE) was used for all three techniques (Supplementary methods).

### Statistical analysis

Patient characteristics and outcomes were compared using a t-test or one-way ANOVA for parametric data, a Mann–Whitney U test or Kruskal–Wallis test for nonparametric data, and Chi-square test for categorical data, stratified by cluster. The primary outcome was all-cause mortality at 28 days, the secondary outcome was all-cause mortality at 60 days. To analyse the association of vilobelimab and mortality between the different clinical clusters, patients were categorized based on their randomization arm (vilobelimab or placebo). The treatment effect was analyzed assessing the interaction term between vilobelimab and mortality using Cox regression if proportional hazards were met, otherwise a logistic regression was employed. To adjust for confounding, age and sex were included in the analysis. Survival was visualized using Kaplan–Meier curves. A *p* value of < 0.05 was considered of statistical significance.

## Results

After randomization, 368 patients received vilobelimab (n = 177) or placebo (n = 191). The median age was 58 years (IQR 47–68) and 252 (68%) were male. All-cause mortality at 28 days was 31% in the vilobelimab group and 40% in the placebo group (HR 0.73 [0.50–1.06], *p* = 0.094) [1].

### Latent class analysis

**Fig. 1:**
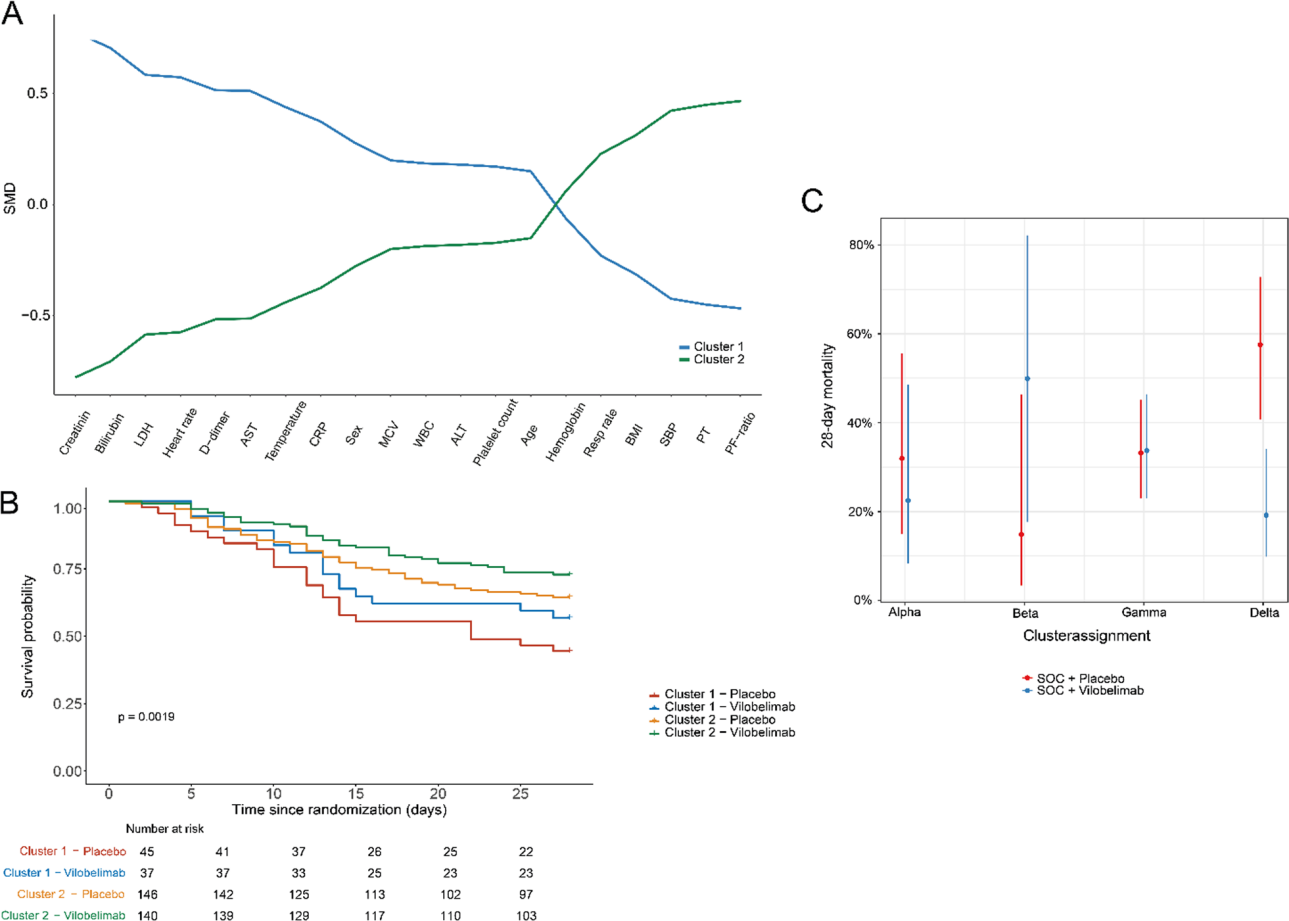
Heterogeneity of treatment effect using different cluster techniques. **A** Profile plot of the two classes identified by LCA using clinical data. All variables used are plotted on the x-axis, with the y-axis displaying standardized mean differences. **B** Kaplan–Meier curves for 28-day mortality of the two classes identified by LCA per treatment group. **C** Heterogeneity of treatment effect of the previously identified clinical sepsis phenotypes, adjusted for age and sex, with in phenotype δ an increase in effect of vilobelimab compared to placebo. Abbreviations: ALT, alanine transaminase; AST, aspartate aminotransferase; BMI, body mass index; CRP, C-reactive protein; LDH, lactate dehydrogenase; MCV, mean corpuscular volume; PF ratio, PaO2/FiO2 ratio; PT, prothrombin time; SBP, systolic blood pressure; SOC, standard of care; WBC, white blood count

After excluding correlated variables (hematocrit, neutrophils and red blood cell count, Supplementary Fig. 1), 20 variables were used as input for LCA. Based on multiple indices, a 2-class latent model was deemed most suitable (Supplementary Table 2). In the 2-class LCA model, 82 (22%) patients were assigned to class 1 and 286 (78%) to class 2 (Table 1). Class assignment did not differ between imputation sets (94.6–98.1% agreement). Class 1 was defined by more severely ill patients, reflected by, among other variables, higher creatinine (120 vs. 77 μmol/L, *p* < 0.001) and bilirubin (12 vs. 8 μmol/L, *p* < 0.001) and lower systolic blood pressure (113 vs. 120 mmHg, *p* = 0.003) and PF-ratio (78 vs. 113, *p* = 0.001). Mortality was significantly higher in class 1 compared to class 2 (28-day mortality 50 vs. 32%, *p* = 0.003, Table 1). In a logistic regression, since the assumption of proportional hazards were not met, adjusted for age and sex, no HTE between classes was observed for 28-day mortality (*p* = 0.998, Fig. 1B) or 60-day mortality (*p* = 0.853). When comparing related Treatment emergent adverse events (TEAE), any or severe, no significant differences were found between the two classes (*p* = 1.000 and *p* = 1.000). The 2-class LCA model showed no clear overlap with the SENECA subtypes, except for a higher proportion of patients with the δ-subtype in Class 1 (Supplementary Table 3). The 4-class latent model was also assessed, because of the higher entropy and significant LMR-LRT p-value, but showed no HTE (*p* = 0.128–0.135) and there was no overlap with the SENECA subtypes (*p* = 0.411, Supplementary Table 4).

**Table 1:** Baseline characteristics and outcome of LCA classes and clinical sepsis phenotypes

	Class 1	Class 2	*p* value	α	β	δ	γ	*p* value
n	82	286		41	17	112	198	
Vilobelimab (%)	37 (45.1)	140 (49.0)	0.627	18 (43.9)	7 (41.2)	57 (50.9)	95 (48.0)	0.809
Demographics								
Sex = Male (%)	64 (78.0)	188 (65.7)	0.048	30 (73.2)	9 (52.9)	93 (83.0)	120 (60.6)	< 0.001
Age (median [IQR])	59 [48, 68]	58 [46, 68]	0.468	52 [46, 62]	63 [57, 73]	60 [47, 68]	58 [46, 67]	0.004
Medical History								
Hypertension (%)	50 (61.0)	122 (42.6)	0.011	14 (34.1)	15 (88.2)	63 (56.2)	80 (40.4)	0.001
Diabetes (%)	27 (32.9)	83 (29.0)	0.665	9 (22.0)	11 (64.7)	26 (23.2)	64 (32.3)	0.022
Chronic Heart Disease (%)	10 (12.2)	20 (7.0)	0.218	3 (7.3)	2 (11.8)	9 (8.0)	16 (8.1)	0.936
COPD (%)	2 (2.4)	6 (2.0)	0.800	2 (4.9)	0 (0.0)	1 (0.9)	5 (2.5)	0.708
Carcinoma (%)	2 (2.4)	3 (1.0)	0.355	1 (2.4)	0 (0.0)	0 (0.0)	4 (2.0)	0.767
Chronic Kidney Disease (%)	6 (7.3)	19 (6.6)	0.681	0 (0.0)	3 (17.6)	10 (8.9)	12 (6.1)	0.123
Obesity (%)	40 (50.0)	162 (57.4)	0.291	27 (69.2)	11 (68.8)	57 (52.3)	107 (54.0)	0.192
Disease Severity								
ARDS (%)			0.013					0.042
Mild	0 (0.0)	2 (0.7)		0 (0.0)	0 (0.0)	1 (0.9)	1 (0.5)	
Moderate	50 (61.0)	218 (76.2)		27 (65.9)	15 (88.2)	70 (62.5)	156 (78.8)	
Severe	32 (39.0)	66 (23.1)		14 (34.1)	2 (11.8)	41 (36.6)	41 (20.7)	
WHO Score (%)			0.334					0.997
6	25 (30.5)	106 (37.1)		15 (36.6)	6 (35.3)	49 (34.8)	71 (35.9)	
7	57 (69.5)	180 (62.9)		26 (63.4)	11 (64.7)	73 (65.2)	127 (64.1)	
Outcome								
28-day Mortality (%)	41 (50.0)	90 (31.5)	0.003	11 (26.8)	6 (35.3)	45 (40.2)	69 (34.8)	0.485
60-day Mortality (%)	45 (54.9)	104 (36.4)	0.004	15 (36.6)	7 (41.2)	49 (43.8)	78 (39.4)	0.836

Abbreviations: ARDS, acute respiratory distress syndrome; COPD, chronic obstructive pulmonary disease; WHO, world health organisation

### Ward’s hierarchical clustering

Using a different algorithm, HC did not result in significant classes (Supplementary Table 5), therefore no further analyses were executed.

### Adjudication of previously identified clinical subtypes

Using the SENECA subtypes, 41 patients (11%) were adjudicated to α, 17 (5%) β, 112 (30%) δ and 198 (54%) subtype γ (Table 1, Supplementary Table 6). In line with previous reports, the δ-subtype was most severely ill, with the highest aspartate aminotransferase (AST) (62, vs α 51, β 35 and γ 39, *p* < 0.001) and C-reactive protein (CRP) (115, vs α 35, β 109 and γ 107, *p* < 0.001, Table 1). Both 28-day and 60-day mortality did not differ between the four subtypes (*p* = 0.485 and *p* = 0.836). Logistic regression, adjusted for age and sex, detected HTE for 28-day mortality (*p* = 0.001, Fig. 1C) and 60-day mortality (*p* = 0.006). Treatment with vilobelimab in the δ subtype was associated with improved 28-day mortality (OR = 0.17 (95% CI 0.07–0.40); *p* < 0.001) and 60-day mortality (OR 0.21 (0.09–0.48); *p* < 0.001). Of note, no signal for harm or benefit was seen in treating patients with vilobelimab in any other clinical subtype (*p* = 0.115–0.790). When comparing related TEAE, any or severe, no significant differences were found between the four subtypes (*p* = 0.685 and *p* = 0.796).

## Discussion

In this secondary analysis of a phase 3 randomized trial, treatment effect with vilobelimab was consistent across different classes and subtypes in critically ill COVID-19 patients, except for a strong effect in the δ subtype. These data suggest potential benefit for the most severely ill patients, with no signal of greater adverse events from vilobelimab in subtypes of critically ill COVID-19 patients.

Our results extend the pre-specified subgroup analysis in the PANAMO trial [1], stratifying patients based on World Health Organisation (WHO) severity score. Treatment effect was most apparent in most severely ill patients; WHO severity score 7 and the δ subtype. In similar work evaluating HTE in the immunomodulation of COVID-19 patients, blood immune endotypes derived from whole-blood mRNA also had HTE for anakinra [13]. Not all approaches are the same, however, as no HTE was observed in newly developed subphenotypes using LCA or hierarchical clustering in our analysis. The results of this study show that based on clinical variables these patients are quite homogenous. This is also in line with previous results based on plasma biomarkers [14]. Surprisingly, HTE was present in phenotypes derived before the existence of COVID-19. First, this means that it is possible for studies to miss HTE when using their own data, possibly because it doesn’t contain enough information. Second, this highlights that using previous phenotypes can be helpful and that phenotypes can be identifiable in other diseases/syndromes than the original population. Overall, this emphasizes that personalized medicine is important in COVID-19 patients, but cluster analysis and developing new phenotypes is not always necessary in an already quite homogenous group of patients.

This study has several strengths and limitations. First, the use of randomised group allocation eliminates selection bias. Second, by using two different forms of unsupervised learning, our findings are more robust. As for limitations, the sample size in some of the classes and subtypes was too small to make reliable inferences. Second, 13 of the 29 variables needed for the adjudication of the previously identified clinical sepsis subtypes were not available in this cohort; however, the distribution of the subtypes was in line with a previous study applying these subtypes in COVID-19 patients with similar variables [5]. Third, the LCA model did not reach an entropy of 0.8, indicating modest class separation. Fourth, no analysis of functional outcomes or long-term mortality beyond 60 days was done.

In conclusion, treatment effect with vilobelimab was consistent across different classes and phenotypes in critically ill COVID-19 patients, except for the δ subtype, where benefit may be present for the most severely ill patients.

### Supplementary Information

Additional file 1 (DOCX 156 kb)

## Data Availability

Data for the completed PANAMO trial will be shared according to applicable regulatory requirements.

## Abbreviations

AST: Aspartate aminotransferase
C5a: Complement 5a
CRP: C-reactive protein
ED: Emergency department
HC: Hierarchical clustering
HTE: Heterogeneity of treatment effect
LCA: Latent class analysis
M3C: Monte Carlo reference-based consensus clustering
PF-ratio: PaO_2_/FiO_2_ ratio
SARS-CoV-2: Severe acute respiratory syndrome coronavirus 2
TEAE: Treatment emergent adverse events
WHO: World Health Organisation
