# Supplementary material for: Heterogeneity of treatment effect of vilobelimab in COVID-19: a secondary analysis of a randomised controlled trial
Source: Crit Care. 2024 Jun 28;28:210. doi: 10.1186/s13054-024-05004-z (PMC11214248; doi:10.1186/s13054-024-05004-z)
Supplement: Supplementary file 1 — Additional file 1 (DOCX 156 kb) [file 13054_2024_5004_MOESM1_ESM.docx]

**Supplement**

**Heterogeneity of treatment effect of vilobelimab in COVID-19: a secondary analysis of a randomised controlled trial**

Rombout B.E. van Amstel^*^, Marleen A. Slim^*^, Endry H.T. Lim, Simon Rückinger, Christopher W. Seymour, Bruce P. Burnett, Lieuwe D.J. Bos, Lonneke A. van Vught, Niels C. Riedemann, Diederik van de Beek, Alexander P.J. Vlaar, on behalf of the PANAMO study group

**Both authors contributed equally.*

| **Content** | **Page** |
| --- | --- |
| Supplementary Methods | 2 |
| Supplementary Table 1. Percentage of missing variables before imputation and transformation | 3 |
| Supplementary Table 2. Model-fit statistics for different number of latent classes  Supplementary Table 3. Confusion matrix of LCA classes and SENECA subtypes  Supplementary Table 4. Baseline characteristics and outcome of four class LCA model | 4  4  5 |
| Supplementary Table 5. Model-fit statistic for Hierarchical clustering | 6 |
| Supplementary Table 6. Variables needed for adjudication of clinical sepsis phenotypes | 7 |
| Supplementary Figure 1. Correlation plot of variables used in latent class analysis | 8 |

**Supplementary Methods**

The four analytical steps that were done before cluster analysis and latent class analysis are listed below. First, correlation between variables was first assessed. If a Spearman correlation coefficient > 0.6 was present, one of the two variables was excluded from clustering based on clinical importance. Second, missing clinical variables were imputed using multivariate imputation by chained equations (MICE) algorithm with predictive mean matching with estimates combined of five imputed datasets with 25 iterations each (1). Third, the variables were transformed to resemble normally distributed data, which was verified using Shapiro-Wilk tests and Q-Q plots. Fourth, the variables were standardized.

Latent class analysis was executed using five sequential models consisting of 1 to 5 classes. As described (2), the best-fitting model was selected based on the Bayesian information criterion, the Lo–Mendell–Rubin adjusted likelihood ratio test (3), class probability, class size and entropy. Once the best model was selected, an individual patient’s class assignment was determined by the highest probability of class membership. Subtype characteristics were visualized using a profile plot, which displayed the standardized mean difference (SMD) of phenotype defining variables.

To assign the SENECA subtypes, all variable units underwent conversion to match the specific units utilized in the SENECA database, and normalization was applied as necessary. Any missing data was imputed following the previously described method. Subsequently, distances to SENECA subtype centroids were computed for each patient in relation to all SENECA subtype centroids. This computation was conducted after scaling all variables using the respective Z-scores from the SENECA derivation cohort. Finally, subjects were categorized into a SENECA subtype based on the minimal overall Euclidean distance observed.

**Supplementary Table 1. Percentage of missing variables before imputation and transformation**

| **Variables** | **Missing %** | **Transformation** |
| --- | --- | --- |
| Age | 0.0 | - |
| ALT | 1.4 | log_10_(x) |
| AST | 1.6 | log_10_(x) |
| Bilirubin | 0.8 | log_10_(x) |
| BMI | 1.6 | log_10_(x) |
| CRP | 1.4 | √x |
| Creatinine | 0.3 | log_10_(x) |
| D-dimer | 6.3 | log_10_(x) |
| Heart rate | 3.5 | log_10_(x) |
| Hemoglobin | 0.3 | log_10_(x) |
| LDH | 4.6 | log_10_(x) |
| MCV | 3.0 | - |
| Neutrophils | 3.5 | - |
| PaO2 | 0.0 | log_10_(x) |
| PF-ratio | 0.0 | log_10_(x) |
| Platelets | 0.3 | - |
| PT | 14.4 | 1/x |
| Respiratory rate | 4.1 | - |
| Sex | 0.0 | - |
| Systolic Blood Pressure | 3.5 | - |
| Temperature | 5.7 | - |
| WBC count | 0.3 | log_10_(x) |

Abbreviations: ALT, alanine transaminase; AST, aspartate aminotransferase; BMI, body mass index; CRP, C-reactive protein; LDH, lactate dehydrogenase; MCV, mean corpuscular volume; PF ratio, PaO2/FiO2 ratio; PT, prothrombin time; SBP, systolic blood pressure; WBC, white blood cell.

**Supplementary Table 2. Model-fit statistics for different number of latent classes**

| **Classes** | **Number of Patients Per Class** | **BIC** | **LMR-LRT** | **LMR-LRT p-value** | **Entropy** |
| --- | --- | --- | --- | --- | --- |
| 1 | 386 | 20512.54 | - | - | - |
| 2 | 82, 286 | 20431.63 | 220.518 | <0.001 | 0.70 |
| 3 | 44, 184, 140 | 20457.45 | 171.678 | <0.001 | 0.76 |
| 4 | 101, 31, 132, 104 | 20492.75 | 177.359 | <0.001 | 0.84 |
| 5 | 105, 72, 48, 27, 116 | 20570.12 | 171.751 | <0.001 | 0.84 |

Abbreviations: BIC, Bayesian information criterion; LMR-LRT, Lo–Mendell–Rubin adjusted likelihood ratio test.

**Supplementary Table 3. Confusion matrix of LCA classes and SENECA subtypes**

|  | **SENECA subtype - α** | **SENECA subtype - β** | **SENECA subtype - γ** | **SENECA subtype - δ** |
| --- | --- | --- | --- | --- |
| **LCA - class 1** | **4 (9.8%)** | **2 (11.8%)** | **33 (16.7%)** | **43 (38.4%)** |
| **LCA - class 2** | **37 (90.2%)** | **15 (88.2%)** | **165 (83.3%)** | **69 (61.6%)** |

**Supplementary Table 4. Baseline characteristics and outcome of four class LCA model**

|  | **Class 1** | **Class 2** | **Class 3** | **Class 4** | ***p*-value** |
| --- | --- | --- | --- | --- | --- |
| **n** | **116** | **98** | **47** | **107** |  |
| Vilobelimab (%) | 65 (56.0) | 45 (45.9) | 23 (48.9) | 44 (41.1) | 0.157 |
|  |  |  |  |  |  |
| **Demographics** |  |  |  |  |  |
| Sex = male (%) | 85 (73.3) | 49 (50.0) | 39 (83.0) | 79 (73.8) | <0.001 |
| Age (median [IQR]) | 65.00 [59.00, 70.25] | 64.00 [58.00, 72.00] | 57.00 [47.00, 66.50] | 40.00 [33.00, 47.00] | <0.001 |
|  |  |  |  |  |  |
| **Medical history** |  |  |  |  |  |
| Hypertension (%) | 60 (51.8) | 60 (61.2) | 31 (66.0) | 21 (19.6) | <0.001 |
| Diabetes (%) | 38 (32.8) | 44 (44.9) | 17 (36.2) | 11 (10.3) | <0.001 |
| Chronic Heart Disease (%) | 10 (8.6) | 16 (16.3) | 4 (8.5) | 0 (0.0) | 0.002 |
| COPD (%) | 5 (4.3) | 2 (2.0) | 1 (2.1) | 0 (0.0) | 0.447 |
| Carcinoma (%) | 2 (1.8) | 2 (2.0) | 1 (2.1) | 0 (0.0) | 0.582 |
| Chronic Kidney Disease (%) | 8 (6.9) | 13 (13.2) | 3 (6.4) | 1 (0.9) | 0.047 |
| Obesity (%) | 47 (41.2) | 53 (55.2) | 22 (46.8) | 80 (76.2) | <0.001 |
|  |  |  |  |  |  |
| **Disease severity** |  |  |  |  |  |
| ARDS (%) |  |  |  |  | 0.084 |
| Mild | 1 (0.9) | 1 (1.0) | 0 (0.0) | 0 (0.0) |  |
| Moderate | 82 (70.7) | 79 (80.6) | 27 (57.4) | 80 (74.8) |  |
| Severe | 33 (28.4) | 18 (18.4) | 20 (42.6) | 27 (25.2) |  |
| WHO score (%) |  |  |  |  | 0.091 |
| 6 | 27 (23.3) | 31 (31.6) | 8 (17.0) | 18 (16.8) |  |
| 7 | 89 (76.7) | 67 (68.4) | 39 (83.0) | 89 (83.2) |  |
|  |  |  |  |  |  |
| **SENECA subtype** |  |  |  |  | 0.411 |
| α | 15 (12.9) | 9 (9.2) | 5 (10.6) | 12 (11.2) |  |
| β | 3 (2.6) | 8 (8.2) | 4 (8.5) | 2 (1.9) |  |
| γ | 62 (53.4) | 56 (57.1) | 23 (48.9) | 57 (53.3) |  |
| δ | 36 (31.0) | 25 (25.6) | 15 (31.9) | 36 (33.6) |  |
|  |  |  |  |  |  |
| **Outcome** |  |  |  |  |  |
| 28-day Mortality (%) - Placebo | 22 (43.1) | 23 (43.4) | 13 (54.2) | 19 (30.2) | 0.128 |
| 28-day Mortality (%) - Vilobelimab | 15 (23.1) | 19 (42.2) | 12 (52.2) | 8 (18.2) |  |
| 60-day Mortality (%) - Placebo | 27 (52.9) | 25 (47.2) | 14 (58.3) | 21 (33.3) | 0.135 |
| 60-day Mortality (%) - Vilobelimab | 21 (32.3) | 21 (46.7) | 12 (52.2) | 8 (18.2) |  |

Abbreviations: ARDS, acute respiratory distress syndrome; COPD, chronic obstructive pulmonary disease; WHO, world health organisation. *P*-value of 28-day and 60-day mortality is the interaction term using a logistic regression to assess HTE.

**Supplementary Table 5. Model-fit statistic for Hierarchical clustering**

| **Classes** | **Monte Carlo p-value** | **Beta Distribution p-value** | **RCSI (95% CI)** |
| --- | --- | --- | --- |
| 2 | 0.96 | 0.89 | -0.054 (-0.071; -0.037) |
| 3 | 0.85 | 0.90 | -0.030 (-0.039; -0.021) |
| 4 | 0.69 | 0.67 | -0.009 (-0.017; -0.001) |
| 5 | 0.96 | 0.97 | -0.036 (-0.043; -0.028) |

Abbreviations: RCSI, Relative Cluster Stability Index.

**Supplementary Table 6. Variables needed for adjudication of clinical sepsis phenotypes**

| **Variables needed** | **Variable Available in PANAMO Cohort** |
| --- | --- |
| Age | Yes |
| Albumin | No |
| ALT | Yes |
| AST | Yes |
| Bands | Yes, neutrophils |
| Bicarbonate | No |
| Bilirubin | Yes |
| BUN | No |
| Chloride | No |
| CRP | Yes |
| Creatinine | Yes |
| Elixhauser | No |
| ESR | No |
| GCS | No |
| Sex | Yes |
| Glucose | No |
| Heart rate | Yes |
| Hb | Yes |
| INR | No |
| Lactate | No |
| Oxygen saturation | No |
| PaO2 | Yes |
| Platelets | Yes |
| Respiratory rate | Yes |
| Sodium | No |
| Systolic Blood Pressure | Yes |
| Temperature | Yes |
| Troponin | No |
| WBC count | Yes |

Abbreviations: ALT, alanine transaminase; AST, aspartate aminotransferase; BUN, blood urea nitrogen; CRP, C-reactive protein; ESR, erythrocyte sedimentation rate; GCS, glascow coma scale; Hb, Hemoglobin; INR, international normalized ratio; WBC, white blood cell.

**Supplementary Figure 1. Correlation plot of variables used in latent class analysis**


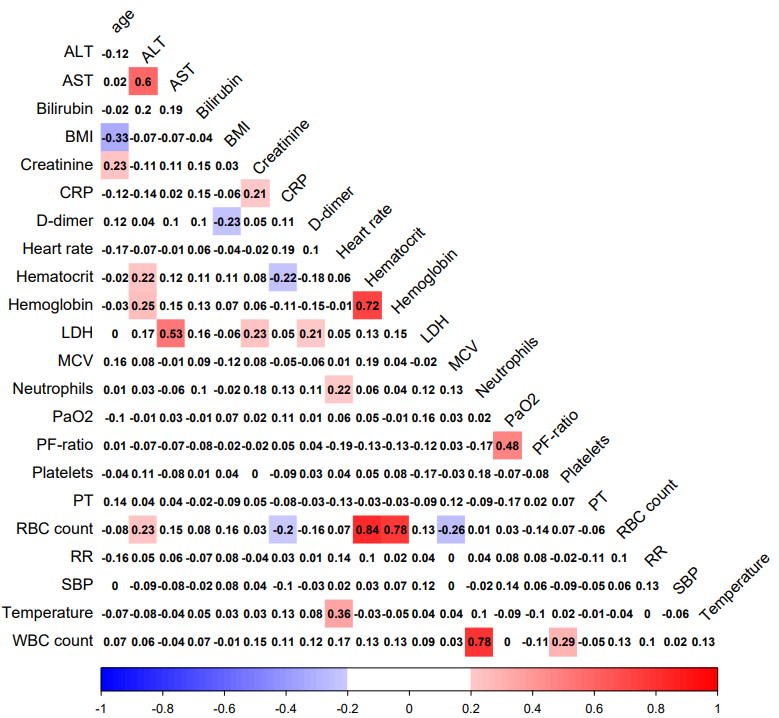


The boxes indicate the Pearson correlation coefficient and are colored according to the scale below. Abbreviations: ALT, alanine transaminase; AST, aspartate aminotransferase; BMI, body mass index; CRP, C-reactive protein; LDH, lactate dehydrogenase; PT, prothrombin time; MCV, mean corpuscular volume; RR, respiratory rate; SBP, systolic blood pressure; WBC, white blood cell.

**References**

1. van Buuren S G-OK. mice: Multivariate Imputation by Chained Equations in R. J Stat Softw. 2011;45(3):1–67.

2. Sinha P, Calfee CS, Delucchi KL. Practitioner's Guide to Latent Class Analysis: Methodological Considerations and Common Pitfalls. Crit Care Med. 2021;49(1):e63-e79.

3. Nylund KL, Asparouhov T, Muthén BO. Deciding on the Number of Classes in Latent Class Analysis and Growth Mixture Modeling: A Monte Carlo Simulation Study. Structural Equation Modeling: A Multidisciplinary Journal. 2007;14(4):535-69.
